# Mass Spectrometry Approaches for SARS-CoV-2 Detection: Harnessing for Application in Food and Environmental Samples

**DOI:** 10.3390/v14050872

**Published:** 2022-04-22

**Authors:** Esaú Bojórquez-Velázquez, Miriam Livier Llamas-García, José M. Elizalde-Contreras, Jesús Alejandro Zamora-Briseño, Eliel Ruiz-May

**Affiliations:** 1 Red de Estudios Moleculares Avanzados, Instituto de Ecología A. C., Cluster BioMimic^®^, Carretera Antigua a Coatepec 351, Xalapa, Veracruz CP 91073, Mexico; jose.elizalde@inecol.mx (J.M.E.-C.); alejandro.zamora@inecol.mx (J.A.Z.-B.); 2 IPICYT, Instituto Potosino de Investigación Científica y Tecnológica A. C., Camino a la Presa San José 2055, San Luis Potosí, San Luis Potosí CP 78216, Mexico; miriam.llamas@ipicyt.edu.mx

**Keywords:** mass spectrometry, SARS-CoV-2, clinical samples, foods, sewage water

## Abstract

The public health crisis caused by the emergence of the Severe Acute Respiratory Syndrome Coronavirus 2 (SARS-CoV-2) in 2019 has drastically changed our lifestyle in virtually all contexts around the world. SARS-CoV-2 is mainly airborne, transmitted by the salivary droplets produced when infected people cough or sneeze. In addition, diarrhea symptoms and the detection of SARS-CoV-2 in feces suggest a fecal–oral route of contagion. Currently, the high demand for SARS-CoV-2 diagnosis has surpassed the availability of PCR and immunodetection probes and has prompted the development of other diagnostic alternatives. In this context, mass spectrometry (MS) represents a mature, robust alternative platform for detection of SARS-CoV-2 and other human viruses. This possibility has raised great interest worldwide. Therefore, it is time for the global application of MS as a feasible option for detecting SARS-CoV-2, not only in human fluids, but also in other matrices such as foods and wastewater. This review covers the most relevant established methods for MS-based SARS-CoV-2 detection and discusses the future application of these tools in different matrices. Significance: The Coronavirus Disease 2019 (COVID-19) pandemic highlighted the pros and cons of currently available PCR and immunodetection tools. The great concern over the infective potential of SARS-CoV-2 viral particles that can persist for several hours on different surfaces under various conditions further evidenced the need for reliable alternatives and high-throughput methods to meet the needs for mass detection of SARS-CoV-2. In this context, MS-based proteomics emerging from fundamental studies in life science can offer a robust option for SARS-CoV-2 detection in human fluids and other matrices. In addition, the substantial efforts towards detecting SARS-CoV-2 in clinal samples, position MS to support the detection of this virus in different matrices such as the surfaces of the packing food process, frozen foods, and wastewaters. Proteomics and mass spectrometry are, therefore, well positioned to play a role in the epidemiological control of COVID-19 and other future diseases. We are currently witnessing the opportunity to generate technologies to overcome prolonged pandemics for the first time in human history.

## 1. Introduction

During the Coronavirus Disease 2019 (COVID-19) pandemic, we witnessed the mass scale use of multiple molecular tools for detection of the Severe Acute Respiratory Syndrome Coronavirus 2 (SARS-CoV-2), which at the same time led to shortages and lack of capacity to meet the sudden, high, and worldwide demand for those tools. The pros and cons of the current methods and the difficulties related to scaling them up to cover the high diagnostic demand were evident. Many studies focused on SARS-CoV-2 detection in human fluids to diagnose infected people with the primary goal of tracking and regulating the epidemiological dynamics of the pandemic [1,2,3]. In addition, strategies were proposed to monitor SARS-CoV-2 titer dynamics in cities by analyzing environmental samples such as sewage water effluents [4]. It is also worth considering other matrices, like food surfaces such as fresh fruit and vegetables and processed foods including sausages, cheeses, and packaged foods as a good alternative for detecting SARS-CoV-2 in a population with active mobility [5,6,7].

Pioneering studies used direct observation methods based on electron microscopy to diagnose and typify foodborne gastroenteric viruses. However, these approaches usually have low sensitivity and specificity, as well as being laborious and poorly scalable [8]. Subsequently, enzyme-linked immunosorbent assay (ELISA) became the primary method for detecting group A rotavirus, adenovirus, astrovirus, and some norovirus (NoV). Currently, the standard methods and techniques for identifying viruses are based primarily on detecting and quantifying viral RNA or DNA by RT-PCR or PCR [9,10]. These techniques are the current gold standard for efficiently detecting SARS-CoV-2 in clinal samples. However, there are issues with these approaches that can decrease certainty in their results. For example, they do not allow the differentiation of infective versus non-infective viral particles. In addition, the propensity of RNA to quickly degrade can give false negatives as a function of the time since sampling and lack of recognition by the oligonucleotides used or nonspecific amplification [11]. The fact that RT-PCR is time consuming and uses costly reagents are further factors that underscore the need for effective alternatives for SARS-CoV-2 detection, especially for non-clinical uses such as environmental monitoring of samples such as foods, surfaces, drinking water, or sewage water effluents. Surprisingly, minimal efforts have focused on standardizing SARS-CoV-2 detection in these matrices.

Amongst the variety of contexts in which SARS-CoV-2 occurrence and persistence continues to be important, the food distribution chain is one the most relevant and undoubtedly one of the most strongly affected sectors by the COVID-19 pandemic. Food could be screened from production through distribution channels to its destination during its handling in markets and supermarkets. The most significant risk of virus transmission by food is through the intake of raw or fresh products. Another risk of contagion is associated with contamination after processing, especially for foods that are refrigerated or frozen, which prolongs the duration of infectivity of the viruses [12]. To date, there are no reports on transmission or contagion of SARS-CoV-2 through food consumption, including foods of animal origin such as poultry, red meat, or fish, which can represent a source of potential risk. However, we cannot rule out the possibility of SARS-CoV-2 or future emerging viruses by food consumption. Thus, we should maintain strict sanitary measures to infections by other viruses that are known to be dispersed through food [13,14]. Therefore, the availability of analytical tools to detect SARS-CoV-2 is of great importance for the proper surveillance and regulation of good handling, processing, packaging, transportation practices, and water management to safeguard food and water supplies during current and future pandemic conditions.

In this context, mass spectrometry (MS) is a robust technology that could aid in the detection of SARS-CoV-2 in food and environmental samples. Furthermore, additional information provided by MS tools (posttranslational modifications) underpin the use of this versatile technology [15]. Access to MS in universities and research institutions could help detect SARS-CoV-2, well beyond clinical laboratories and medical institutions. For this reason, in this review, we present the different approaches through which MS has contributed tools that help combat the current pandemic and possible future health contingencies and suggest the alternative use of mass spectrometry for SARS-CoV-2 detection in a variety of matrices.

## 2. Virus Detection by Mass Spectrometry-Based Approaches

The most widely used mass spectrometric approach for pathogen identification is matrix-assisted laser desorption ionization-time of flight mass spectrometry (MALDI-TOF MS) [16,17,18,19]. The success of this platform is due to the multiple in vitro protocols developed for bacteria biotyping methods, many of which have been approved by regulatory agencies such as the US Food and Drug Administration (FDA). However, using MALDI-TOF to direct viruses has drawbacks, especially the insufficient sensitivity to detect the low amount of viral protein in samples. Moreover, the most abundant proteins in viral particles are high-mass molecules (>20 kDa), which significantly reduces sample ionization. Furthermore, MALDI pipelines require the establishment of pathogen cultures prior to pathogen identification, which delays analysis and exponentially increases the difficulty of the process, due to the carry-over of contamination from the culture media [17].

The introduction of Electrospray ionization (ESI) in proteomic studies provided new advantages over MALDI. ESI outperforms MALDI in most cases because the liquid chromatographic system allows the separation of complex mixtures of peptides, which allows more time for the MS to generate more spectra and therefore provides more information per sample. In addition, ESI enables the establishment of quantification techniques such as selected reaction monitoring (SRM) and parallel reaction monitoring (PRM), providing quantification of peptides and proteins at the attomole level [20]. Despite limitations, both MALDI-TOF and liquid chromatography (LC)-MS/MS have been reliably used for virus detection in epidemiological studies of different strains from complex mixtures in clinical and environmental samples. For instance, studying in vitro cultures allowed the detection of murine hepatitis virus (MHV) and influenza A virus strains [21,22]. Studies have shown that the LC-MS/MS approach allows identifying monoculture viruses to discriminate between two types of viruses in the same sample, which is not possible using MALDI-TOF MS due to its low sensitivity. Different strains of influenza A virus cultured in LLC-MK2 cells—eight H1N1 and two H3N2—were identified and adequately differentiated at the type and subtype level with a viral load limit of 1 × 10^9^ and 7 × 10^6^ copies of the viral genome by MALDI-TOF MS and LC-MS/MS, respectively, through peptides derived only from nucleocapsid protein (N). The LC-MS/MS approach also differentiated between viruses of different species present in the same sample, which was evaluated using mixtures of influenza H3N2 and RSV (Respiratory Syncytial Virus) and influenza H1N1 and hMPV (human metapneumovirus). The detection thresholds were 3.6 × 10^7^ and 1.1 × 10^7^ copies for RSV and hMPV, respectively [21]. The influenza viruses were classified as subtypes in both mixtures with a detection threshold equivalent to ≥5.3 × 10^7^ copies of the viral genome. The identification of the four viruses was based on nucleocapsid protein-derived peptides. In addition to N peptides, viral matrix protein 1 (M1), non-structural protein 1 (NS1), hemagglutinin (HA), and neuraminidase (NA) peptides were also identified for influenza. For RSV and hMPV, peptides were also identified for matrix protein (M), matrix protein 2-1 (M2-1), phosphoprotein (P), and fusion glycoprotein (F) [21].

Although there has been success in implementing MS-based techniques to use proteins as a virus detection target, diagnosis using these biomolecules has benefits and disadvantages. The stability of proteins is advantageous over the instability of nucleic acids, especially RNA. MS would, therefore, provide evidence of chains of contagion by exposure as a function of time by monitoring the transit history of food, for example. One of the main advantages of MS approaches over nucleic acid-based methods is that exponential amplification of the analyte is not required, which eliminates a potential source of false positives. Since MS tools allow the detection of structural protein modifications, this technology can provide additional information to understand the biology of the pathogen of interest [23]. Nevertheless, the direct detection of viruses by MS requires ultrasensitive techniques and expensive equipment due to the minimal amounts of the target molecules detected in the samples [24].

## 3. Mass Spectrometry Approaches for COVID-19 Diagnosis

As mentioned above, mass spectrometry is a promising approach as an analytical tool for fast, accurate virus detection from different matrices. Prior to the COVID-19 pandemic, the outbreaks of Severe Acute Respiratory Syndrome Coronavirus (SARS-CoV) in 2003 and Middle East Respiratory Syndrome Coronavirus (MERS-CoV) in 2012 prompted initiatives to implement MS-based detection protocols for human coronaviruses (HCoVs). One example of these protocols was the combination of multiplex RT-PCR and MALDI-TOF. Xiu et al. used this approach to detect six HCoVs: HCoV-229E, HCoV-OC43, HCoV-NL63, HCoV-HKU1, SARS-CoV, and MERS-CoV. Specific amplicons for the RNA-dependent RNA polymerase (RdRp) and N genes were analyzed by MS, achieving detection limits of 10–100 copies without cross-reactivity with other respiratory viruses [25]. These MS strategies represent an alternative for diagnosis, but the main disadvantages are the availability of specialized equipment and the need for lengthy sample preparation steps, which increases the analysis time. In an ideal scenario, pathogen detection and diagnostic analyses should require a reduced number of processing steps and minimal manipulation of the sample to make the generation of results more efficient, reducing costs without compromising the accuracy of the result.

Shortly after announcing the state of pandemic due to the infectious agent SARS-CoV-2, the scientific community developed alternative diagnostic and prognostic techniques and methods to help solve the growing demand due to the increasing number of cases every day. Despite these efforts, the analytical capacity of countries worldwide to carry out diagnostic tests was exceeded, especially during surges and peaks of contagion, which generally occurred after holidays. The mass spectrometry community reacted to this contingency, aiming to make sample collection and processing MS protocols available to the community to minimize the damage caused by COVID-19 disease [26]. In the last two years, the efforts to establish detection methods for SARS-CoV-2 proteins has led to the establishment of multiple MS-based alternatives that differ in aspects including ionization source, mass analyzer, sample origin, processing, and target peptides analysis.

The first report of SARS-CoV-2 protein identification using MS for possible diagnostic applications was by Gouveia et al. [27]. These authors used Vero E6 cells infected with the virus and in-gel digestion coupled with nanoLC-MC/MS, allowing the identification of 101 peptides corresponding to six viral proteins: nucleocapsid protein (N), spike (S), membrane glycoprotein (M), ORF1ab, ORF3a, and ORF8. After establishing exclusion parameters suitable for a targeted approach, the number of peptides was reduced to 14, belonging to the structural viral proteins M, N, and S. Finally, when considering inter- or intraspecies specificity criteria, presence of modifications, and detection of missed proteolytic cleavages, only four peptides were the best candidates, LQSLQTYVTQQLIR, FQTLLALHR, HTPINLVR from S protein, and VAGDSGFAAYSR from M protein. Some peptides from N protein also were suggested as possible targets due to their specificity for SARS-CoV-2 (ADETQALPQR, AYNVTQAFGR, NPANNAAIVLQLPQGTTLPK, and WYFYYLGTGPEAGLPYGANK) [27]. This work also endorses the importance of using controlled laboratory conditions and in vitro experiments (such as the use of cell lines) to standardize detection methods prior to determining SARS-CoV-2 in real-life samples. For example, before implementing proteomics-based diagnostic tests, we should consider parameters including the stage of the disease in patients, type of matrix, or environmental samples, which could have a significantly lower number of copies of viral proteins than in vitro cell cultures.

Several works have addressed the challenge of analyzing clinical samples to offer a diagnostic alternative. The first one was reported by Ihling et al. [28]. In this study, by using gargle solution, it was possible to detect viral nucleocapsid protein-derived peptides in samples with a high viral load (10^5^ to 10^6^ genome equivalents/µL) that had been previously verified by RT-PCR. It is worth noting that the initial sample volume was 20 mL, but only 750 µL were taken for analytical procedures. Although this protocol requires several processing steps and long processing time because it requires overnight precipitation with acetone, it shows the potential for direct SARS-CoV-2 detection using MS-based protein identification for diagnostic purposes, even using a small fraction of the original sample. However, the miniaturization of sample collection and processing will be a bottleneck for generating quality results [28]. Gouveia et al. used an MS-approach for the detection of SARS-CoV-2 on simulated clinical samples [27]. In this work, the authors mixed up increasing amounts of inactivated virus protein hydrolysates (equivalent to 1–544 plaque-forming units, PFU) with hydrolyzed proteins from nasopharyngeal swabs of healthy volunteers. Following this assay, it was possible to identify 18 peptides from structural proteins with the highest virus concentration (544 PFU or 460 ng of protein), including eight peptides from the nucleocapsid protein, seven peptides from the spike protein, and three peptides from the membrane glycoprotein. Only peptides derived from N protein (ADETQALPQR, KADETQALPQR, and GFYAEGSR) were detected in samples with lower amounts of virus (2 and 7 PFU, 2 and 6 ng of protein, respectively). These peptides were also the most consistently detected across the dilutions. However, an option closer to the pandemic conditions would be to homogenize known amounts of inactivated virus in raw nasopharyngeal swab samples taken from healthy patients and follow the processing steps and MS detection. This approach could determine the extractability, hydrolysis, background noise, and detection efficiency of viral proteins. Another goal of the work of Gouveia et al. was to detect SARS-CoV-2 in nasopharyngeal swabs from patients with infections confirmed by RT-PCR [29]. Using in-gel digestion and nanoLC-MS/MS, it was possible to determine five peptides from N protein (LDDKDPNFK, KADETQAIPQR, KKADETQAIPQR, ADETQAIPQR, and GFYAEGSR) and one from the M protein (EITVATSR). The peptides GFYAQGSR and ADETQALPQR exhibited the best values of identification and therefore the highest feasibility as detection biomarkers, suggesting that the simultaneous detection of these two peptides can confer indisputable evidence of the virus presence [29].

Nikolaev et al. evaluated two sample preparation protocols that differed from the final step of sample concentration [30]. First, virus from nasopharyngeal samples was inactivated by heat at 65 °C for 30 min. Then, samples were mixed with isopropanol. In the first approach, the mixture was lyophilized. In the second, the proteins were precipitated by centrifugation of the cooled samples at 20,000× *g* for 20 min. Then, both samples were resuspended and digested with trypsin under the same conditions and analyzed by LC-MS/MS. The centrifugation strategy outperformed the lyophilization method, allowing the identification of an extensive list of peptides associated with the N protein, supporting the frequent detection of RPQGLPNNTASWFTALTQHGK and ADETQALPQR [30].

**Figure 1:**
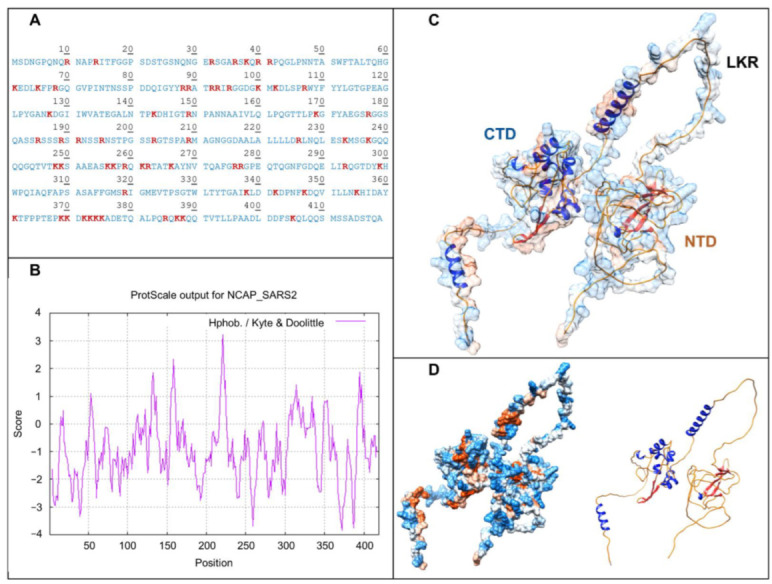
Physicochemical characteristics of SARS-CoV-2 nucleocapsid protein that make it a good candidate for MS processing and analysis. (**A**) Amino acid sequence, the 60 lysine and arginine are highlighted in bold red font; (**B**) Kyte and Doolittle hydrophobicity exhibiting the prevalence of polar regions along the N protein primary structure; (**C**) AlphaFold homology model where NTD, CTD, and LKR are indicated; (**D**) surface and ribbon models that show with detail the exposure of polar amino acid residues and the presence of a high proportion of unstructured regions.

The structural proteins that are incorporated into the final virion, S (spike), E (envelope), M (membrane), and N (nucleocapsid), are the most suitable diagnostic targets due to their high expression and accumulation during infection. The S protein is the largest and could generate more peptides detectable by MS. However, this protein is heavily glycosylated, which affects its fractionation and ionization. The membrane proteins E and M have a shorter sequence, so the possible number of peptides per molecule to be obtained for MS analysis is smaller but are present in higher copy numbers than S; however, their location could decrease their extractability [30]. N protein has a lysine (K) and arginine (R) amount that together account for 14.3% of protein amino acid composition (Expasy ProtParam), which implicates 57 theoretical trypsin cleavage sites (Expasy PeptideCutter) and favor its positive ionization and detection and it is necessary for the ability to bind to nucleic acids (Figure 1A). In addition, the hydropathy profile of SARS-CoV-2 nucleocapsid protein reflects a high content of hydrophilic segments and a Grand average of hydropathicity (GRAVY) of −0.971 (Expasy ProtScale), which suggest the presence of a considerable high proportion of unstructured regions (Figure 1B) [31]. The in silico structural model obtained with AlphaFold shows that the structured N-terminal (NTD) and C-terminal (CTD) domains involved in RNA binding and dimerization are linked by the intrinsically disordered central linker region (LKR) (Figure 1C) [32,33,34]. The surface and ribbon models show the high proportion of exposed polar amino acids and the overall unstructured nature of this protein (Figure 1D). Taken together, the characteristics of N protein could allow an easy extractability and processing, making it an ideal candidate for the implementation of MS diagnostic protocols amongst the structural proteins of the virus.

After the mass of studies attempting to determine the viral peptides that are most likely to be identifiable by MS, there have been works that seek to establish protocols to implement assays directed towards specific peptides. Singh et al. used nanoLC-MS/MS to determine the best SARS-CoV-2 peptides for multiple reaction monitoring methods (MRM) [35]. Peptides such as IVSTIQRKYK from Replicase polyprotein 1ab and QIAPGQTGK from S protein were considered as promising candidates for MRM, due to the consistency of their detection and spectra quality, sensitivity (90.4%), and specificity (100%) of detection with respect to RT-PCR [35]. Zecha et al. carried out a robust proteomics study of SARS-CoV-2 [36]. They evaluated the response to infection in different cell lines and the ability to detect the virus by parallel reaction monitoring (PRM). For this purpose, they used 113 stable isotope-labeled (heavy) peptides that represent all theoretical tryptic peptides covering 11 SARS-CoV-2 proteins. Most of these peptides were detected by MS during their analytical characterization. However, proteomic analysis of proteins from infected Vero E6 cells with SARS-CoV-2 allowed the identification of 57 endogenous viral peptides associated with the biological sample and not with the spiked heavy isotope peptides used. The peptide VAGDSGFAAYSR derived from M protein showed the best overall performance in the PRM assay, and only six unique peptides for SARS-CoV-2 belonging S and ORF9b proteins were identified in clinical samples. However, the successful detection of SARS-CoV-2 was restricted to individuals with very high viral loads [36]. Non-structural proteins were not considered as candidates for detection since they are not part of the virion and are not expressed in large quantities [30]. Nevertheless, proteins may present sufficient levels of detection throughout the infection process. Illustrative examples included the replicase polyprotein 1ab and ORF9b proteins whose peptides are suggested as final targets for diagnosis by MS. Cazares et al. used recombinant versions of proteins N and S to analyze PRM performance in an in vitro mucus culture [20]. The peptides with the best values of detection were DQVILLNK and ADETQALPQR for N, and NIDGYFK and FQTLLALHR for S, with linearities of r2 between 0.995 and 0.986 in a concentration range from 3 amol to 12.5 fmol, while in mock viral samples the limits of detection and quantification were ~200 and ~390 amol, respectively, which is equivalent to 2 × 10^5^ viral particles/mL [20].

The versatility of MS allows the establishment of different approaches for diagnosing COVID-19 beyond protein identification. One method includes amplifying segments of the genetic material using PCR for detection using MALDI-MS. As mentioned before, this type of analysis allowed the detection and differentiation of six human coronaviruses (HCoV-229E, HCoV-OC43, HCoV-NL63, HCoV-HKU1, SARS-CoV, and MERS-CoV), and more recent works have suggested that it is more sensitive than RT-PCR for the detection of SARS-CoV-2 [25,37]. Another strategy was based on the differential phenotype manifested during infection compared with healthy individuals. The diagnosis of diseases does not necessarily require the direct detection/identification of the molecular components of the causative pathogen, but it is also possible to do so through the context of the patient’s symptoms. Based on this, machine learning algorithms were used to classify the contrasting signals generated by MALDI-MS, using the data provided by positive and negative samples for SARS-CoV-2 previously validated by RT-PCR. Two main works have addressed this approach, in the first one Nachtigall et al. incorporates 211 positive and 151 negative nasal swab samples to their study establishing a diagnostic method with up to 93.9% accuracy, 7% false positives, and 5% false negatives [38]. In the second one, Costa et al. used a non-invasive saliva sampling protocol at different stages (0, 10, and 30 days from the time of inclusion in the study) after patients were diagnosed for COVID-19 [39]. The best results were obtained at day 0 with an accuracy of 85.2%, sensitivity of 85.1%, and specificity of 85.3%, evaluated in a population of 105 positive and 51 negative patients for SARS-CoV-2 [39]. The potential application of this alternative approach has the advantage that the sample is applied directly to the MALDI plate for analysis without prior processing, considerably reducing diagnostic times and costs. Furthermore, the availability of the MALDI-TOF platform even in developing countries supports the global accessibility of this pipeline.

## 4. Perspectives on the Implementation of SARS-CoV-2 MS-Based Detection in Environmental Samples

MS provides an alternative for virus detection with high confidence and resolution. We underline the existence of robust and mature MS-based detection protocols for SARS-CoV-2 (Table 1) and other viruses. Although most recent efforts have been oriented to SARS-CoV-2, we consider that it is time to extend MS-based virus detection as a conventional, routine technology, that can be applied for a variety of samples such as food matrices (e.g., fruit and vegetable surfaces, frozen meat, poultry, fish, and processed foods) and sewage water. Regarding the safe handling of food in the context of the current pandemic, the Food and Agriculture Organization (FAO) has provided two official documents indicating best practices. The first, published in 2012 as part of the CODEX ALIMENTARIUS, entitled *“GUIDELINES ON THE APPLICATION OF GENERAL PRINCIPLES OF FOOD HYGIENE TO THE CONTROL OF VIRUSES IN FOOD”*, establishes the hygiene regulations to be followed for all types of food, with an emphasis on ready-to-eat foods, from primary production to the consumer to control enteric viruses. These rules were developed to trace and control the increasing incidence of infections caused by foodborne viruses, mainly NoV and hepatitis A virus (HAV), and to a lesser extent other viruses such as rotavirus, hepatitis E virus (HEV), astrovirus, Aichi virus, sapovirus, enterovirus, various types of coronaviruses, parvovirus, and adenovirus (though the list could be even longer [40]). In the second document, *“COVID-19 and food safety: guidance for food businesses”*, published on 7 April 2020, the FAO established that since the food industry personnel, involved in the production and preparation of food, cannot carry out their tasks from home and it is necessary to maintain the continuous supply of food, food companies must urgently implement the measures required to prevent COVID-19 contagion amongst workers, control their exposure and transmission, and strengthen food hygiene and sanitation practices [41]. The cleanliness of the facilities and work equipment is an essential factor, as SARS-CoV-2 can be stable and maintain its contagion capacity on different surfaces: up to 72 h on plastic and stainless steel, 4 h on copper, and 24 h on cardboard. Moreover, the environmental stability of SARS-CoV-2 variants of concern can also vary considerably; Omicron is the most stable with 21.1 and 193.5 h on human skin and plastic surfaces, respectively [42,43,44]. Currently, there are no studies about SARS-CoV-2 viability on food surfaces, rather only on food packaging, which can be a point of contagion as a function of time. This aspect is particularly important for foods that require cold chain maintenance since SARS-CoV-2 can be more stable when refrigerated (4 °C) or frozen (−10 to −80 °C). The need for low temperature provoked infection points related to the fresh food trade, mainly seafood, including significant outbreaks at the end of 2019 in Wuhan and in June 2020 in Beijing. Regular SARS-CoV-2 detection and fast identification of SARS-CoV-2-infected individuals amongst food industry workers may be warranted [45]. Another critical point of control that should be constantly monitored is sewage water, since, as previously mentioned, SARS-CoV-2 is also present in the feces of infected individuals. An increase in the presence of viral RNA in wastewater correlated with an increase in COVID-19 prevalence [46]. Once SARS-CoV-2 reaches pluvial distribution networks and sewer systems, the pathogenic agent can spread, mainly in regions where wastewater is used to irrigate crops for human consumption, suggesting a fecal–oral route of transmission for COVID-19 [43]. In fact, it has been suggested that analyzing sewage water from campus dorms in universities can be used as surveillance strategy for early detection of SARS-CoV-2 [4]. Establishing sampling and analysis protocols for the detection of SARS-CoV-2 in sewage systems could therefore form part of an epidemiological surveillance strategy to support decision making in health institutions, define action plans, and inform communication directed to public to reduce the rate of contagion [47,48].

The main challenges to overcome in virus detection in different sample matrices is the scalability of sample processing and automatization of the analysis for a massive number of samples. In other cases, the matrix composition can reduce the detection limit of the target protein or peptide. For example, human fluids such as saliva and blood contain high-abundance proteins, increasing the fluid proteome’s dynamical range, and masking possible viral proteins [49,50,51]. Therefore, efficient protocols for depleting highly abundant proteins will be critical to overcoming these current limitations. In addition, heavily glycosylated proteins in human fluids hinder proper protein digestion, make chromatographic separation difficult, reduce column lifespan, and decrease the optimum behavior of LC-MS/MS-based methods. In this context, mixed proteases could provide additional digestion of glycoproteins, including those of viral origin. In addition, microwave-assisted digestion showed excellent results reducing sample preparation time and obtaining better protein coverage [52]. Foods are even more complex matrices; for example, in red meat, poultry, and fish, the abundance of animal proteins can interfere. In contrast, in the case of plants, metabolites such as pigments, sugars, lipids, or waxes in the surface of fruits and vegetables make it necessary to establish cleaning stages before analysis by mass spectrometry. The availability of different resins for solid phase peptide extraction and protocols such as filter aided sample preparation (FASP) and combinatorial peptide ligand library (CPLL) could provide an alternative to digging deep in the proteome, allowing the confident detection of peptides or proteins associated with SARS-CoV-2 [53,54]. It is worth noting that innovation in terms of reagents, protein digestion, and peptide fractionation has been minimal after several years. We expect to see more top-down approaches compared to bottom-up proteomics pipelines not only on plant biology but also in human fluid proteomics. In wastewater, the primary constraint is the lower viral load in large volumes; therefore, sample concentration by lionization and filtration could be the key for detecting SARS-CoV-2 [55]. Filter paper and centrifuge filter devices with various capacities and molecular weight cutoffs may provide an alternative for fast viral protein concentration, complementing a precipitation protocol. Regardless of the approach used, methods must be fully validated, like any other technique intended to be used as a common standard. Multiple protocols can be used as complementary approaches, providing global accessibility of reliable testing in current and future pandemic situations.

## 5. Conclusions

Mass spectrometry provides a wide range of tools that can be used to prevent the damage caused by both the current pandemic and possible future health contingencies. We therefore consider that clinical diagnostic methods based on proteomics and mass spectrometry can soon be translated to other targets of epidemiological monitoring, such as food, beverages, or wastewater. The most promising mass spectrometry methods are SRM and PRM, which have allowed the generation of an extensive list of candidate peptides as excellent targets for directly detecting viral proteins and have proven their feasibility for SARS-CoV-2 detection at very low concentrations. In addition, the versatility of mass spectrometry platforms will allow global access to efficient detection of SARS-CoV-2 and other viruses.

## Figures and Tables

**Table 1:** Proposed peptides as candidates to be used in diagnostic methods for SARS-CoV-2/COVID-19 through targeted proteomics.

Reference	Sample	Protein	Identified Peptides	General Protein Processing	Ionization/MS Platform
Gouveia et al. [27]	Vero E6 cells	M	VAGDSGFAAYSR	SDS-PAGE-Shotgun	nanoLC-Q-Exactive HF
		N	ADETQALPQR AYNVTQAFGR NPANNAAIVLQLPQGTTLPK WYFYYLGTGPEAGLPYGANK		
		S	LQSLQTYVTQQLIR FQTLLALHR HTPINLVR		
Ihling et al. [28]	Gargle solution	N	RPQGLPNNTASWFTALTQHGK	Acetone precipitation-Shotgun	nanoLC-Orbitrap Fusion Tribrid
Gouveia et al. [29]	Simili /Nasopharyngeal	N	ADETQALPQR	SDS-PAGE-Shotgun	nanoLC-Q-Exactive HF
			GFYAEGSR		
Nikolaev et al. [30]	Nasopharyngeal	N	ITFGGPSDSTGSNQNGER RPQGLPNNTASWFTALTQHGK GQGVPINTNSSPDDQIGYYR WYFYYLGTGPEAGLPYGANK DGIIWVATEGALNTPK NPANNAAIVLQLPQGTTLPK MAGNGGDAALALLLLDR MAGNGGDAALALLLLDRLNQLESK RGPEQTQGNFGDQELIR GPEQTQGNFGDQELIR IGMEVTPSGTWLTYTGAIK LDDKDPNFK LDDKDPNFKDQVILLNK KADETQALPQR ADETQALPQR KQQTVTLLPAADLDDFSK QQTVTLLPAADLDDFSK QLQQSMSSADSTQA	Isopropoanol precipitation-Shotgun	nanoLC-timsTOF Pro
Singh et al. [35]	Nasopharyngeal and	N	ADETQALPQR	TCA precipitation-	nanoLC-TripleTOF 6600
	oropharyngeal	Replicase 1ab	AIVSTIQRKYK	Shotgun	QTrap 6500+
			LTDNVYIK		
			MDGSIIQFPN		
		S	LIANQFNSAIGK		
			STNLVKNK		
			AHFPREGVFVSNGTHWFVTQR		
			QIAPGQTGK		
Zecha et al. [36]	Vero E6 cells/	M	EITVATSR	SDS-PAGE-Shotgun	nanoLC-Fusion Lumos Tribrid
	Nasopharyngeal		LNTDHSSSSDNIALLVQ		
			VAGDSGFAAYSR		
		N	ADETQALPQR		
			AYNVTQAFGR		
			GFYAEGSR		
			QQTVTLLPAADLDDFSK		
			GQGVPINTNSSPDDQIGYYR		
			IGMEVTPSGTWLTYTGAIK		
			NPANNAAIVLQLPQGTTLPK		
		S	KVGGNYNYLYR		
			RFASVYAWNR		
			RVQPTESIVR		
			RVVVLSFELLHAPATVCGPK		
			FLPFQQFGR		
			GIYQTSNFR		
			LQSLQTYVTQQLIR		
			VYSTGSNVFQTR		
		ORF8	LGSLVVR		
		ORF9b	LGSPLSLNMAR		
			LVDPQIQLAVTR		
			LATTEELPDEFVVVTVK		
			VYPIILR		
Cazares et al. [20]	In vitro derived mucus	N	DQVILLNK	Shotgun	nanoLC-Q-Exactive HF-X
			ADETQALPQR		
		S	NIDGYFK		
			FQTLLALHR		

## Data Availability

Not applicable.
